# Concurrent acute myocardial infarction and acute ischemic stroke in a diabetic patient undergoing chemotherapy for non-Hodgkin lymphoma: Should I administer thrombolytic therapy? A case report

**DOI:** 10.1186/s43044-024-00593-0

**Published:** 2024-12-25

**Authors:** Sigfrid Casmir Shayo, Khuzeima Khanbai, Yona Gandye, Flora Lwakatare, Nakigunda Kiroga, Tatizo Waane, Peter Kisenge

**Affiliations:** 1 Jakaya Kikwete Cardiac Institute, P.O. Box 65141, Dar es Salaam, Tanzania; 2 Department of Radiology, Muhimbili National Hospital, P.O. Box 65000, Dar es Salaam, Tanzania

**Keywords:** Ischemic stroke, ST-elevation myocardial infarction, Cardio-cerebral infarction, CHOP chemotherapy, Percutaneous coronary intervention, Thrombolysis, Endovascular mechanical embolectomy, Case report

## Abstract

**Background:**

Concurrent ST-elevation myocardial infarction (STEMI) and acute ischemic stroke (AIS) are extremely rare, and their management remains perplexing due to the absence of high-quality evidence and limited resources. For the first time, we report a rare, preventable, and suboptimally managed case of concurrent AIS and STEMI in a patient with non-Hodgkin lymphoma (NHL) who received cyclophosphamide, doxorubicin, vincristine, and prednisolone (CHOP) chemotherapy.

**Case presentation:**

A 59-year-old postmenopausal woman of African origin with a background history of type 2 diabetes mellitus presented to the Jakaya Kikwete Cardiac Institute with sudden onset of left-sided weakness and typical ischemic chest pain for 3 days. The patient was recently diagnosed with NHL and started CHOP chemotherapy 3 weeks prior. Physical examination revealed left-sided hemiplegia. Emergency brain computed tomography and 12-lead echocardiography (ECG) revealed AIS and STEMI, respectively. A diagnosis of concurrent AIS and STEMI was reached, and the patient was loaded with dual antiplatelets and heparin and rushed for emergency coronary angiography (GAG) and percutaneous coronary intervention (PCI). CAG revealed massive thrombotic occlusion of the mid-segment of the left anterior descending coronary artery (mLAD) and proximal segment of the right coronary artery. Revascularization was achieved in both vessels with a resultant TIMI flow grade of 3. The post-PCI period was marked by significant improvement in chest pain and resolution of ST-elevation, as revealed by 12-lead ECG. However, the patient remained hemiplegic.

**Conclusion:**

We have described a rare case of concurrent AIS and STEMI in a postmenopausal woman who had a significant risk of thromboembolism. The patient had uncontrolled type 2 diabetes and received CHOP chemotherapy for NHL, which was diagnosed 3 weeks prior. This case underscores the need for thromboembolic prophylaxis for selected cancer patients receiving chemotherapy. The need to individualize management is also emphasized, as both PCI and thrombolysis carry the risk of serious repercussions. In our patient, if thrombolysis was attempted it would have caused myocardial rupture and immediate death. The patient would have benefited from endovascular mechanical embolectomy for AIS; however, this practice is lacking at our institution. This calls for the establishment and strengthening of neurointerventional practices in our tertiary healthcare facilities.

## Background

There has been an alarming global increase in the incidence of cardiovascular disease and its traditional risk factors, particularly in middle- and low-income countries [1]. Traditional risk factors for cardiovascular disease include visceral obesity, metabolic syndrome, type 2 diabetes mellitus (T2DM), and smoking. All of these factors orchestrate chronic systemic low-grade inflammation that is responsible for atherosclerosis of coronary, cerebral, and peripheral arteries, which is referred to as atherosclerotic cardiovascular disease (ASCVD) [2, 3].

Acute myocardial infarction (AMI) and stroke are the two major vascular events with a significant toll on healthcare expenditure, quality-adjusted life years, morbidity, and mortality [4]. These catastrophic vascular events share common triggering factors, including oxidative stress, inflammation, and endothelial dysfunction(s) [5].

Although these events share important risk factors as well as triggering factors, concurrent AMI and AIS are rare findings. In 2010, Omar and his colleagues used the term cardio-cerebral infarction to refer to simultaneous AMI and AIS. The incidence of cardio-cerebral infarction is reported to be as low as 0.009% [6, 7].

There has never been a reported case of concurrent AIS and AMI occurring in a patient who received cancer chemotherapy, specifically CHOP chemotherapy. We present a rare, preventable, and suboptimally managed case of concurrent AIS and AMI occurring in a postmenopausal woman who received chemotherapy for NHL.

## Case presentation

A 59-year-old postmenopausal woman with a 5-year history of type 2 diabetes mellitus presented to the Jakaya Kikwete Cardiac Institute with sudden onset of left-sided weakness and excruciating chest pain that had worsened over 3 days. The patient was recently diagnosed with non-Hodgkin lymphoma and started chemotherapy with CHOP 3 weeks prior. She denied prior hypertension or heart disease but reported using a menopausal supplement.

Physical examination revealed a conscious patient who experienced chest pain with left-sided hemiplegia but no facial asymmetry or aphasia. She was hemodynamically stable with unremarkable vital signs.

**Fig. 1:**
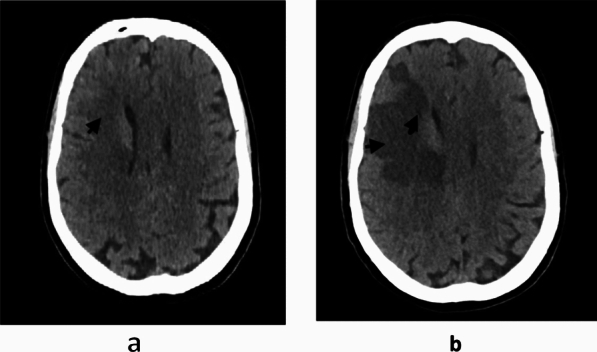
**a** Brain CT scan taken 8 h after left-sided hemiplegia; the arrow indicates early ischemic changes. **b** Control brain CT scan taken 72 h after left-sided hemiplegia; arrows indicate extensive cortical infarct

**Fig. 2:**
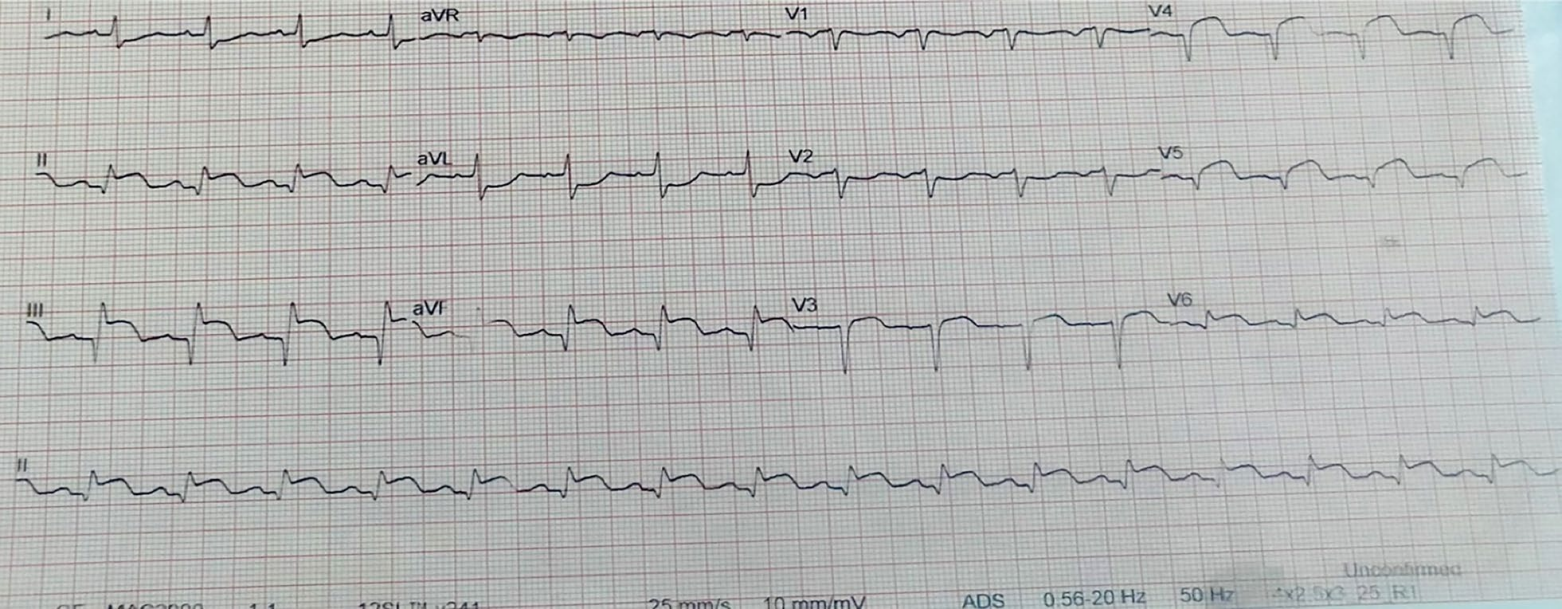
Patient 12-lead ECG at presentation

An emergency CT scan revealed early ischemic stroke features (Fig. 1a), which were later confirmed by a follow-up CT scan showing an extensive infarct (Fig. 1b). A 12-lead ECG showed significant ST-segment elevation in the inferior leads, i.e., leads II, III, and aVF, with reciprocal changes in leads I and aVL. There was also some ST-segment elevation in leads V5 and V6 (Fig. 2). Blood tests revealed markedly elevated troponin I, C-reactive protein (CRP), glycated hemoglobin (HbA1c), and random blood glucose (RBG).

**Fig. 3:**
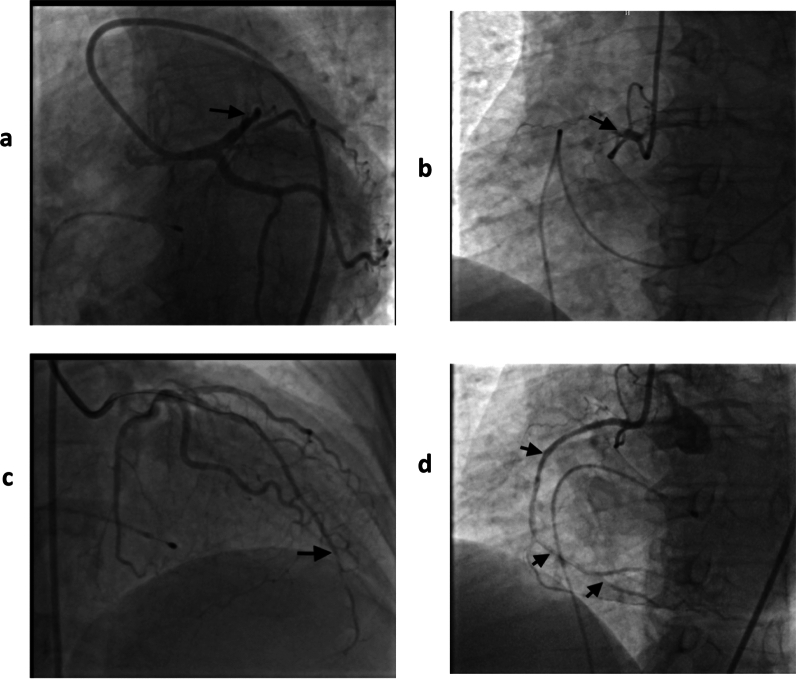
**a** Coronary angiography of a patient at presentation; the arrow indicates an occluded mLAD. **b** Coronary angiography of the patient at presentation; arrow shows a completely occluded pRCA. **c** Coronary angiography of the patient after balloon angioplasty of the mLAD; the arrow depicts good distal flow. **d** Coronary angiography of the patient after stenting of the pRCA. Arrows indicate good flow to the middle and distal segments of the RCA

A diagnosis of concurrent acute myocardial infarction (AMI) and acute ischemic stroke (AIS) was made. The patient was loaded with clopidogrel 600 mg, aspirin 300 mg, and atorvastatin 80 mg, and prepared for emergency coronary angiography (CAG) and percutaneous coronary intervention (PCI). CAG revealed total thrombotic occlusion of the mid-segment of the left anterior descending coronary artery (mLAD) (Fig. 3a) and proximal segment of the right coronary artery (pRCA) (Fig. 3b). Balloon angioplasty of the mLAD and stenting of the pRCA were successfully carried out. TIMI flow grade 3 was achieved in both vessels (Fig. 3c, d).

The patient developed ventricular fibrillation during coronary angiography, which was treated with defibrillation, but later developed bradycardia requiring temporary pacing. Following PCI, she was admitted to the coronary care unit (CCU) and received anticoagulation therapy. Her chest pain improved, but she developed a throbbing headache on the third day, which was ruled out as hemorrhagic transformation of the ischemic stroke (Fig. 1b).

She was transferred to the high-dependency unit (HDU) and continued on dual antiplatelet therapy, heparin, furosemide, and atorvastatin. She showed remarkable improvement and was discharged after 9 days in the hospital. 12-lead ECG done before the patient was discharged showed normal sinus rhythm with complete resolution of ST-segment elevation. Echocardiography revealed ischemic cardiomyopathy with reduced left ventricular systolic function (LVEF = 34%).

At discharge, the patient had stable vital signs but residual left-sided hemiplegia. She was advised to continue dual antiplatelet therapy, rivaroxaban, and metformin–glimepiride combination therapy and was scheduled to visit a neurology clinic. Her oncology team planned to continue chemotherapy after her discharge from the hospital.

### Patient’s perspective

“I was not sure if I could survive until this time. Three days before suffering left side paralysis, I started feeling pain in my chest, left arm, and left shoulder. The pain was worsening and, despite taking over-the-counter pain medication, it did not improve. It was not until the day that I suffered left side paralysis that I was rushed to this cardiac centre.

I thank the doctors for the life-saving procedure. The pain has gone, but my left side is still weak. I can’t lift or walk. I also have hard and scanty stools. Since I started using chemotherapy 3 weeks ago, I have started feeling bad. My blood glucose readings were also high. I think chemotherapy has brought all this suffering.”

## Conclusions

To the best of our knowledge, this is the first case of concurrent AMI and AIS following diagnosis of NHL and initiation of chemotherapy. According to the American Society of Hematology, the diagnosis of cancer increases the incidence of stroke and AMI. Patients with incident cancer have a substantially short-term risk of arterial thromboembolism [8]. This situation is further aggravated by the use of certain chemotherapies, such as doxorubicin, which have been shown to also increase the risk of thromboembolism [9]. Our patient had received CHOP chemotherapy with doxorubicin as one of the components approximately three weeks prior to the onset of acute cardio-cerebral infarction. As the patient’s symptoms and worsening health conditions are linked with the initiation of chemotherapy, it is more likely that in addition to uncontrolled T2DM, the initiation of chemotherapy might have contributed to acute cardio-cerebral infarction. Antithrombotic therapy is generally recommended for the prevention of both arterial and venous thromboembolism in high-risk cancer patients, particularly those treated with chemotherapy [10]. In contrast, our patient did not receive a single antiplatelet or anticoagulant prior to having a cardio-cerebral infarction. Therefore, there is a need to increase awareness among oncologists about the risk of thrombosis among cancer patients with comorbidities undergoing chemotherapy.

It is worth recognizing that inflammation is at the heart of vascular endothelial dysfunction and thromboembolism. Our patient had a remarkably elevated high-sensitivity CRP (106 mg/dL) but a normal CBC at presentation. Her HbA1c and RBG levels were 11.76% and 18 mmol/L, respectively, indicating poorly controlled T2DM. This finding suggested that sterile inflammation induced by danger-associated molecular patterns (DAMPs) from killed cancer cells as well as uncontrolled T2DM contributed to the occurrence of arterial thrombosis.

The diagnosis of cardio-cerebral infarction exerts significant decision-making pressure on attending clinicians. This is because urgent action is needed to restore perfusion to the myocardium as well as the brain. Concurrent AMI and AIS are very rare clinical conditions for which there is no high-quality evidence or clinical guidelines for management. This patient landed on the best facility for cardiovascular care in the country with a state-of-the-art catheterization laboratory and highly experienced interventional cardiologists. We were therefore biased to serve the myocardium first. According to the European Society of Cardiology 2023 guidelines for the management of acute coronary syndrome (ACS), an invasive strategy should be undertaken to reperfuse the myocardium in cancer patients presenting with ACS with an expected survival of > 6 months [11]. The decision to perform PCI in this patient was reached but with significant worries about hemorrhagic transformation following the administration of anticoagulation and a high dose of dual antiplatelets, which is a requirement for performing PCI. Although the patient did not develop hemorrhagic transformation of the AIS, the infarct size increased, and she remained hemiplegic. Owing to the risk of myocardial rupture [12], our patient was not eligible for thrombolytics that would concurrently help to reperfuse the brain and myocardium. The patient would likely benefit from endovascular mechanical embolectomy for AIS; however, this practice is lacking at our institution. This calls for the establishment and strengthening of neurointerventional practices in our tertiary hospitals.

Upon discharge, the patient presented with hemiplegia, remained hemodynamically stable, and exhibited a significant reduction in left ventricular systolic function (EF = 34%). Two days later, she proceeded with the second cycle of chemotherapy. The patient's prognosis is guarded, with favorable outcomes reliant upon patient-centered multidisciplinary collaboration among oncologists, cardiologists, and neurologists. Additionally, the patient's ability to cover healthcare expenses and adhere to prescribed therapy is crucial.

In conclusion, heightened awareness is necessary among oncologists regarding the importance of administering prophylactic antithrombotic medications to high-risk newly diagnosed cancer patients undergoing chemotherapy due to the increased risk of arterial thrombosis. Managing cardio-cerebral infarction in patients with comorbidities poses significant challenges, particularly in resource-limited settings. In the absence of robust evidence and guidelines, treatment approaches must be highly individualized. Thus, it is imperative to establish and maintain advanced multidisciplinary practices and foster collaboration among experts to achieve optimal treatment outcomes.

## Data Availability

Not applicable.

## Abbreviations

CHOP: Cyclophosphamide, doxorubicin, vincristine, prednisolone
ASC: Acute coronary syndrome
AIS: Acute ischemic stroke
AMI: Acute myocardial infarction
STEMI: ST-elevation myocardial infarction
CAG: Coronary angiography
PCI: Percutaneous coronary intervention
CT: Computed tomography
ECG: Electrocardiography
TIMI: Thrombolysis in myocardial infarction
T2DM: Type 2 diabetes mellitus
ASCVD: Atherosclerotic cardiovascular disease
LAD: Left anterior descending coronary artery
RCA: Right coronary artery
CCU: Coronary care unit
LVEF: Left ventricular ejection fraction
DAMP: Damage-associated molecular patterns
CRP: C-reactive protein
CBC: Complete blood count
RBG: Random blood glucose
HbA1c: Glycated hemoglobin
